# The Rash That Didn’t Blanch: A Case Report of Adult-Onset IgA Vasculitis with Underlying Cirrhosis and IgA Nephropathy

**DOI:** 10.5070/M5.52253

**Published:** 2026-04-30

**Authors:** Elaha Noori, Tyler Rigdon, Omar Darwish, Danielle Matonis

**Affiliations:** * University of California, Irvine, School of Medicine, Irvine, CA; ^ University of California, Irvine, Department of Emergency Medicine, Orange, CA; † University of California, Irvine, Department of Internal Medicine, Orange, CA

## Abstract

Rashes are a common presentation in the emergency department (ED) and can signify a wide variety of underlying conditions. Early recognition of serious dermatologic rashes in the acute care setting is essential in improving patient outcomes. Here we describe the case of a 69-year-old female with a medical history of cirrhosis with ascites, chronic kidney disease (CKD) secondary to immunoglobulin A (IgA) nephropathy, and hypertension who presented to the ED with a chief complaint of foot pain with an associated rash. On examination, she had tender purpura with bilateral symmetric pedal edema. Initial work up in the ED was significant for mildly elevated inflammatory markers. Given the concern for vasculitis, dermatology was consulted; punch biopsy confirmed IgA vasculitis. The patient was subsequently admitted for acute renal injury and started on corticosteroids. After a week-long inpatient stay, she was discharged home in stable condition. This case highlights the importance of maintaining a broad differential diagnosis for purpuric rashes, recognizing when specialty consultation is warranted, and understanding the pathophysiologic interplay between IgA vasculitis, liver cirrhosis, and IgA nephropathy.

**Topics:**

Vasculitis, IgA vasculitis, Henoch-Schönlein purpura, IgA nephropathy, dermatology.

**Figure:**
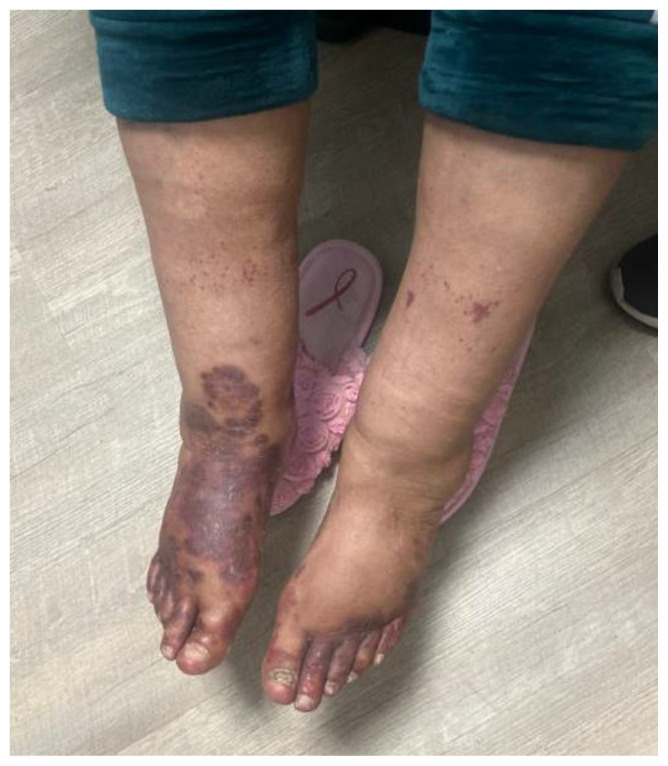


**Figure:**
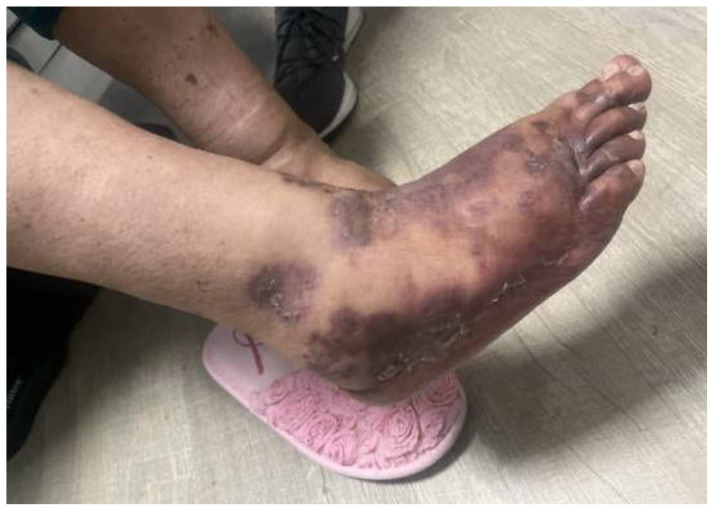


## Brief introduction

Rashes are a frequent presenting complaint in the Emergency Department (ED), accounting for up to 8% of all visits.^1^ The differential diagnosis for a rash is broad, ranging from benign, self-limited etiologies such as contact dermatitis to severe, life-threatening disorders such as toxic epidermal necrolysis. Proper recognition of key clinical features, including lesion morphology, distribution, and associated systemic symptoms, is essential to distinguish benign dermatologic conditions from those requiring prompt intervention. One particularly concerning finding on physical examination is purpura, which is a non-blanching skin lesion due to extravasation of blood into the dermis. The presence of purpura can indicate a variety of serious underlying conditions, including vasculitis, coagulopathies, or life-threatening infections.^2^

Vasculitides encapsulate a wide spectrum of rare conditions characterized by reactive damage and inflammation to blood vessel walls.^3^ Immunoglobulin A (IgA) vasculitis, formerly known as Henoch-Schönlein purpura, is an immune-mediated vasculitis characterized by IgA1 deposits in small arterial walls and nonlymphoid tissue. It primarily affects the small vessels of the skin, kidneys, gastrointestinal tract, and joints. Manifestations in the central nervous system are a less frequent cause.^4^ In the skin, it often presents with non-thrombocytopenic purpura or urticaria.^5^

IgA vasculitis is the most common form of vasculitis in children, with an estimated incidence rate of 3–27 per 100,000 children and approximately 90% of cases occurring in children less than 15 years old.^6^,^7^ In adults, however, IgA vasculitis is exceedingly rare, with reported incidence rates of 0.8–1.8 per 100,000.^5^ While adult-onset IgA vasculitis is typically self-limiting, severe complications such as gastrointestinal perforation, acute kidney injury secondary to IgA nephritis, and circulatory shock may occur.^8^ With patients frequently presenting to the emergency department (ED) with rashes of varying severity, it remains critical for the emergency medicine physician to consider vasculitic disorders in the differential diagnosis of purpura. Here we present the case of a 69-year-old female who presented with bilateral lower extremity purpura and was ultimately found to have IgA vasculitis. This case underscores the importance of maintaining a broad differential when evaluating rashes and highlights the need for vigilance in identifying vasculitides in adult patients.

## Presenting concerns and clinical findings

A 69-year-old female with a past medical history of cirrhosis with ascites, chronic kidney disease (CKD) secondary to IgA nephropathy, and hypertension presented to the ED with a one-day history of foot pain with associated rash. The patient described a rash of abrupt onset with a burning and itchy sensation in her bilateral feet, with right foot pain greater than left. She denied any generalized illness, fevers, chills, abdominal pain, or recent trauma. Upon physical examination, the patient was found to have tender purpura primarily involving the bilateral feet and soles. The lesions were purple, palpable, non-blanching, and tender to palpation, with right foot involvement greater than left. Additionally, there were mild maculopapular lesions and petechiae extending up the calves and mid-thigh, along with bilateral symmetric pedal edema. Vital signs and physical examination were otherwise unremarkable. Written consent was obtained from the patient for photograph and image publication.

Regarding the patient’s past medical history, her stage 2 CKD was confirmed by a renal biopsy in 2021 which had revealed mild IgA nephropathy with hypertensive changes. She had regular follow-up with outpatient nephrology. Her baseline creatinine and estimated glomerular filtration rate (eGFR) levels were 0.92 milligrams per deciliter (mg/dL) and 67 milliliters per minute per 1.73 square meters (mL/min/1.73 m^2^), respectively. Her most recent urine-protein creatinine ratio (uPCR) was measured three months prior to the current presentation and was found to be moderately increased at 260 mg/g.

## Significant findings

The patient’s workup in the ED included labs, computed tomography (CT) imaging, ultrasound imaging, and a dermatology consult. Labs were remarkable for hemoglobin of 9.7 grams per deciliter (g/dL) which was her baseline and a new elevated creatinine at 1.5 mg/dL. The erythrocyte sedimentation rate was not found to be elevated (22 millimeters per hour (mm/h)), but the serum C-reactive protein level was elevated at 3.3 mg/dL. Urinalysis was indicative of pyuria with positive leukocyte esterase, an elevated white blood cell count of 182 per high-power field, proteinuria, microscopic hematuria with red blood cell count of 102 per high-power field, and presence of a few bacteria. Ultrasound and CT angiography of the lower extremities were unremarkable.

Given the patient’s physical exam findings of tender palpable purpura to the lower extremities, dermatology was consulted for suspicion of acute small vessel vasculitis. A punch biopsy for hematoxylin and eosin (H&E) stain and direct immunofluorescence (DIF) was subsequently performed. The H&E demonstrated nonspecific purpura. The DIF, however, showed granular deposits for IgA and C3 (complement component 3) around the superficial perivascular vessels, which is diagnostic of IgA vasculitis.

## Patient course

The constellation of clinical symptoms, physical exam findings, and histologic findings provided strong support for our diagnosis of IgA vasculitis in this patient. Due to the rapid progression of the non-blanching rash and the risk of further kidney damage to the patient, she was subsequently admitted to the hospital with a diagnosis of IgA vasculitis and with dermatology, rheumatology, and nephrology closely following the patient. This decision was further solidified by the dermatology team who also agreed with the plan for admission and further workup.

During the patient’s inpatient course, she was started on prednisone 80 mg daily with plans for future outpatient taper. The patient’s foot burning and itching were treated symptomatically with topical lidocaine cream, topical hydrocortisone cream, and gabapentin 300 mg, the latter of which was renally dosed due to the patient’s renal impairment. The rheumatology team conducted autoimmune serology, which was notable for antinuclear antibodies of 1:160 and a positive antibody to ribonucleoprotein. Tests for human immunodeficiency virus, hepatitis viruses, rheumatoid factor, complement, anti-neutrophil cytoplasmic autoantibodies, and cryoglobulin were negative. These results further provided high clinical support of IgA vasculitis and helped rule out other underlying pathologies.

Furthermore, the patient was also evaluated by the nephrology team. The nephrology team had initially attributed the acute kidney injury to acute tubular necrosis in the setting of contrast-induced nephropathy from the CT angiogram that was done in the ED. However, given that the new elevated creatinine in the ED occurred prior to CT imaging, prerenal factors and underlying chronic kidney disease may have contributed. In fact, the patient’s fractional excretion of sodium (0.1%) and factional excretion of urea (20%) were consistent with prerenal acute kidney injury. The microscopic hematuria seen on urinalysis was also attributed to the patient’s chronic IgA nephropathy rather than acute renal involvement of IgA vasculitis. Serum creatinine peaked at 1.9 mg/dL but then trended down throughout hospitalization to near baseline levels. On hospital admission day 7, the patient was discharged in stable condition with outpatient rheumatology referral and plan to follow-up with her nephrologist. She was advised to continue the lidocaine cream, hydrocortisone cream, gabapentin, and the prednisone taper (80 mg for 5 days, 60 mg for 5 days, 40 mg for 5 days, and finally 20 mg daily until her rheumatology appointment).

## Discussion

In the acute care setting, certain dermatologic findings warrant prompt recognition and evaluation to prevent significant morbidity and mortality. In particular, the presence of non-blanching purpura on physical examination signifies the extravasation of blood into the skin and is often a result of perivascular inflammation, infection, or hematologic dysfunction. It is a concerning finding that can indicate a wide range of benign or life-threatening pathology, including vasculitides, coagulopathies, disseminated intravascular coagulation (DIC), meningococcemia, Rocky Mountain Spotted Fever, endocarditis, and other life-threatening infections.^9^ The presence of a fever or palpable rash can help further narrow the differential diagnosis. Fever with palpable purpura is often associated with infectious causes or IgA vasculitis, while fever with nonpalpable purpura can indicate DIC or thrombotic thrombocytopenic purpura. On the other hand, the lack of fever can indicate autoimmune vasculitis with the finding of palpable purpura or idiopathic thrombocytopenia purpura with the finding of nonpalpable purpura.^10^,^11^ It is therefore critical for the emergency medicine physician to recognize when further evaluation and intervention for purpuric rashes is required because delayed management can have potentially severe sequelae.

This report highlights the case of a 69-year-old female with an acute presentation of IgA vasculitis, which was suspected by the concerning finding of rapid-onset, non-blanching, and palpable purpura upon initial presentation to the ED. IgA vasculitis is an extremely rare and uncommonly reported systemic vasculitis in the adult population, and it most commonly occurs in the pediatric population where its etiology is generally better understood. IgA is a major class of antibody that serves as the first line of defense against mucosal infections, and the deposition of IgA1-dominant immune complexes is central to the disease pathogenesis.^4^ In children, the condition often has an identifiable trigger such as upper respiratory infections, gastrointestinal infections, certain medications, and vaccinations.^11^,^12^ In adults, however, many cases are idiopathic. Moreover, the disease course has also been shown to be more aggressive in adults, with age being a contributing factor for worse outcomes.^13^ For instance, while children commonly present with mild joint and gastrointestinal symptoms, adults present with increased risk of severe purpura and acute renal injury.^14^,^15^ Thus, early diagnosis and treatment are imperative in preventing these complications, and the timely recognition and management of IgA vasculitis in our adult patient are particular strengths of this case report.

Diagnostic criteria for IgA vasculitis are not well-established in adult patients, but the European League Against Rheumatism and the Pediatric Rheumatology European Society diagnostic criteria can help aid diagnosis. These criteria have a 99% sensitivity and 86% specificity in adults, and they include a mandatory criterion of purpura or petechiae in the lower extremities with at least one of the following: arthritis or arthralgia of acute onset, diffuse abdominal pain of acute onset, histopathology showing leukocytoclastic vasculitis or proliferative glomerulonephritis with IgA deposits, and renal involvement with either proteinuria or hematuria.^16^ In our patient, the diagnosis of IgA vasculitis was strongly supported by the clinical presentation of palpable purpura and DIF demonstrating IgA deposits. Secondary etiologies that could justify the occurrence of the patient’s symptoms, such as viral infections and autoimmune diseases, were effectively ruled out which further strengthened the diagnosis.

Since the condition is typically self-limiting in 94% of children and 89% of adults, primary management involves supportive care, including adequate hydration and symptomatic relief of pain.^17^ The role of glucocorticoids in treating IgA vasculitis is questionable because while it may ameliorate the inflammatory process, previous studies in children treated with corticosteroids have shown no significant alterations in the disease course.^18^,^19^ Nevertheless, our patient was still started on a tapered prednisone course to prevent further kidney damage in the context of her pre-existing CKD.

While the exact pathogenesis of IgA vasculitis remains unclear, there have been several studies linking IgA vasculitis to IgA nephropathy due to their shared mechanism of a dysregulated immune response to antigens and IgA deposition.^20^,^21^ In both conditions, IgA1 hinge regions are galactose-deficient, and these modified immunoglobulins are recognized by autoantibodies that create immune complexes and lead to inflammation.^22^ In addition, immunogenetic studies suggest a common genetic susceptibility to both conditions, with specific HLA-DRB1 alleles showing a strong association.^23^ Despite the findings of these studies, there is still no definitive consensus as to whether these are two manifestations of the same condition that occur on a spectrum, especially since there exists no evidence from clinical trials.

Interestingly, abnormalities of IgA have also been noted to arise in advanced liver disease, especially liver cirrhosis. The liver plays an important role in the clearance of intravascular IgA, and liver cirrhosis is linked to IgA metabolism abnormalities. In an experimental study analyzing circulating immune complexes in patients with cirrhosis, Tissandié et al found that these patients had galactose deficiency, increased amounts of abnormally glycosylated polymeric IgA1, and increased CD89 expression on mononuclear cells.^24^ These aberrant IgA1 were found to form complexes with immunoglobulin G and soluble CD89, which is similar to the complexes found in patients with IgA nephropathy. Furthermore, there have also been a few case reports describing the presence of IgA vasculitis in patients with underlying liver cirrhosis, but there is limited analysis of epidemiological data to create a strong association.^25^–^27^ Thus, with all the aforementioned studies in mind, we can speculate that the pathogenic mechanism of our patient with IgA vasculitis may be related to her pre-existing IgA nephropathy and liver cirrhosis, but further clinical studies investigating the association between these conditions would be highly valuable.

Overall, this case exemplifies the importance of rapid identification and timely coordination with multiple specialties in appropriately diagnosing and treating IgA vasculitis in the acute care setting. Although its presentation is rare in adult patients, IgA vasculitis can result in complicated sequalae that require close follow-up, so it is important for the emergency medicine physician to not miss this diagnosis.

## Supplementary Information




