# Supplementary figures and images for: The Rash That Didn’t Blanch: A Case Report of Adult-Onset IgA Vasculitis with Underlying Cirrhosis and IgA Nephropathy
Source: J Educ Teach Emerg Med. 2026 Apr 30;11(2):V10–5. doi: 10.5070/M5.52253 (PMC13152361; doi:10.5070/M5.52253)

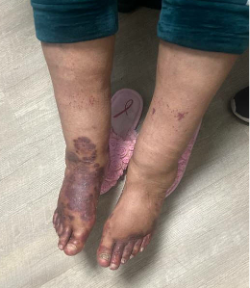

Supplement: Supplementary file 1 [file 11-2-V10-Supp1.png]

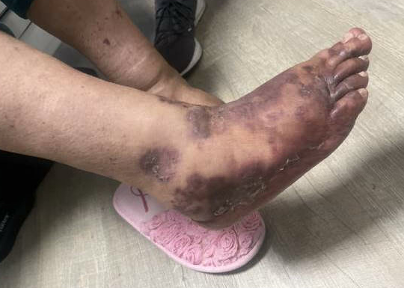

Supplement: Supplementary file 2 [file 11-2-V10-Supp2.png]
